# Prediction of knee osteoarthritis progression using radiological descriptors obtained from bone texture analysis and Siamese neural networks: data from OAI and MOST cohorts

**DOI:** 10.1186/s13075-022-02743-8

**Published:** 2022-03-08

**Authors:** Ahmad Almhdie-Imjabbar, Khac-Lan Nguyen, Hechmi Toumi, Rachid Jennane, Eric Lespessailles

**Affiliations:** 1 EA 4708-I3MTO Laboratory, University of Orleans, Orléans, France; 2 Translational Medicine Research Platform, PRIMMO, Regional Hospital of Orleans, Orléans, France; 3 Department of Rheumatology, Regional Hospital of Orleans, Orléans, France

**Keywords:** Trabecular bone texture, Fractal analysis, Siamese CNNs, Knee osteoarthritis, Subchondral bone, Radiography

## Abstract

**Background:**

Trabecular bone texture (TBT) analysis has been identified as an imaging biomarker that provides information on trabecular bone changes due to knee osteoarthritis (KOA). In parallel with the improvement in medical imaging technologies, machine learning methods have received growing interest in the scientific osteoarthritis community to potentially provide clinicians with prognostic data from conventional knee X-ray datasets, in particular from the Osteoarthritis Initiative (OAI) and the Multicenter Osteoarthritis Study (MOST) cohorts.

**Patients and methods:**

This study included 1888 patients from OAI and 683 patients from MOST cohorts. Radiographs were automatically segmented to determine 16 regions of interest. Patients with an early stage of OA risk, with Kellgren and Lawrence (KL) grade of 1 < KL < 4, were selected. The definition of OA progression was an increase in the OARSI medial joint space narrowing (mJSN) grades over 48 months in OAI and 60 months in MOST. The performance of the TBT-CNN model was evaluated and compared to well-known prediction models using logistic regression.

**Results:**

The TBT-CNN model was predictive of the JSN progression with an area under the curve (AUC) up to 0.75 in OAI and 0.81 in MOST. The predictive ability of the TBT-CNN model was invariant with respect to the acquisition modality or image quality. The prediction models performed significantly better with estimated KL (KLprob) grades than those provided by radiologists. TBT-based models significantly outperformed KLprob-based models in MOST and provided similar performances in OAI. In addition, the combined model, when trained in one cohort, was able to predict OA progression in the other cohort.

**Conclusion:**

The proposed combined model provides a good performance in the prediction of mJSN over 4 to 6 years in patients with relevant KOA. Furthermore, the current study presents an important contribution in showing that TBT-based OA prediction models can work with different databases.

## Background

Knee osteoarthritis (KOA) is a musculoskeletal condition frequently encountered not only in primary care but also in orthopedic and rheumatology clinics [3]. Due to the heterogeneity of osteoarthritis, i.e., its numerous phenotypes [27] and the wide variability in the trajectory of disease progression [12], it is of the utmost importance to identify KOA patients who have a greater potential of progressing more rapidly.

Therefore, it is relevant to develop imaging biomarkers that can help the emergence of new therapeutic treatments and particularly new disease-modifying drugs. Due to the role of the subchondral bone and its remodeling status in KOA progression, texture analysis and tibial subchondral bone mineral density assessments are recognized and established methods to characterize structural alterations associated with KOA [18]. Recently, using the OAI database, the predictive ability of baseline trabecular bone texture to distinguish patients with or without radiographic progression was slightly improved compared to that of conventional clinical risk factors such as age, gender, body mass index (BMI), and joint space width (JSW) [10, 15]. Previously published studies have shown only moderate performance for predicting KOA progression when using pain, race, and previous knee injury [8, 17] as predictor factors. However, since data for pain, race, and previous knee injury were available in both OAI and MOST cohorts, we evaluated the performance of our proposed models with these three additional clinical predictors.

In parallel with the improvement in medical imaging technologies, several machine learning techniques have been proposed for the diagnosis and prediction of KOA [14, 26]. Automatic KOA diagnosis is becoming increasingly popular [4, 23, 26] as it has a high potential to complement the OA diagnostic chain and make radiographic KOA grading more objective.

The aims of this study were twofold: (i) to evaluate the predictive ability of a combined approach using both trabecular bone texture (TBT) descriptors, calculated by a variogram-based method [9, 10], and radiological gravity scores, calculated by deep learning-based Siamese CNN tools [26], to predict KOA progression; (ii) to study the use of the same KOA progression prediction model validated on independent OA cohort datasets (OAI and MOST), by training the model on one dataset and testing it on the other, and vice versa. The TRIPOD checklist (Transparent reporting of a multivariable prediction model for individual prognosis or diagnosis) was used as a framework of quality assurance of the present manuscript [22].

## Methods

### Patients

In this study, the data used in the preparation were obtained from the OAI and the MOST databases. Details about the acquisition and grading protocols in the OAI and the MOST studies are available online at https://nda.nih.gov/oai and http://most.ucsf.edu, respectively. The primary selected dataset included only the knee images of patients with available KL grades [13] and the Osteoarthritis Research Society International (OARSI) grades as well as the clinical covariates: age, gender, BMI, Western Ontario and McMaster universities osteoarthritis index (WOMAC) pain, race, and history of knee injury. From the selected dataset, the knees with preexisting OA with 2 ≤ KL < 4 [5, 10] at baseline were considered in the present study, in accordance with the European Medicines Agency [3] which recommended to include patients with KL radiographic entry criteria of grades 2 or 3 for studies of structure-modifying drugs. The selected dataset was divided into two sub-datasets according to the type of acquisition modality: the computed radiographs (CR), i.e., digital images acquired by a device using X-ray-sensitive plates which are then read by a processor, and the digitized X-ray films (RG).

**Fig. 1:**
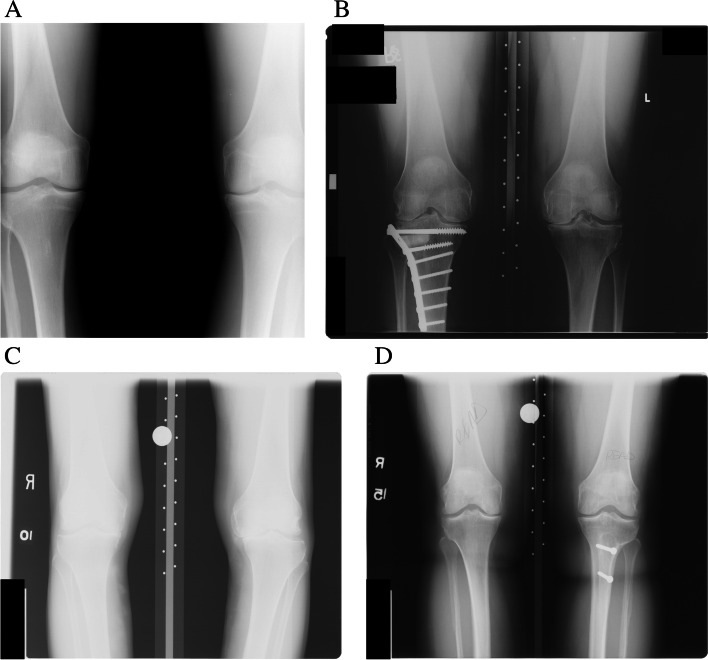
Radiographs from the OAI cohort with overexposure (**A**) and with materials (**B**), and radiographs from the MOST cohort with overexposure (**C**) and with materials (**D**)

**Fig. 2:**
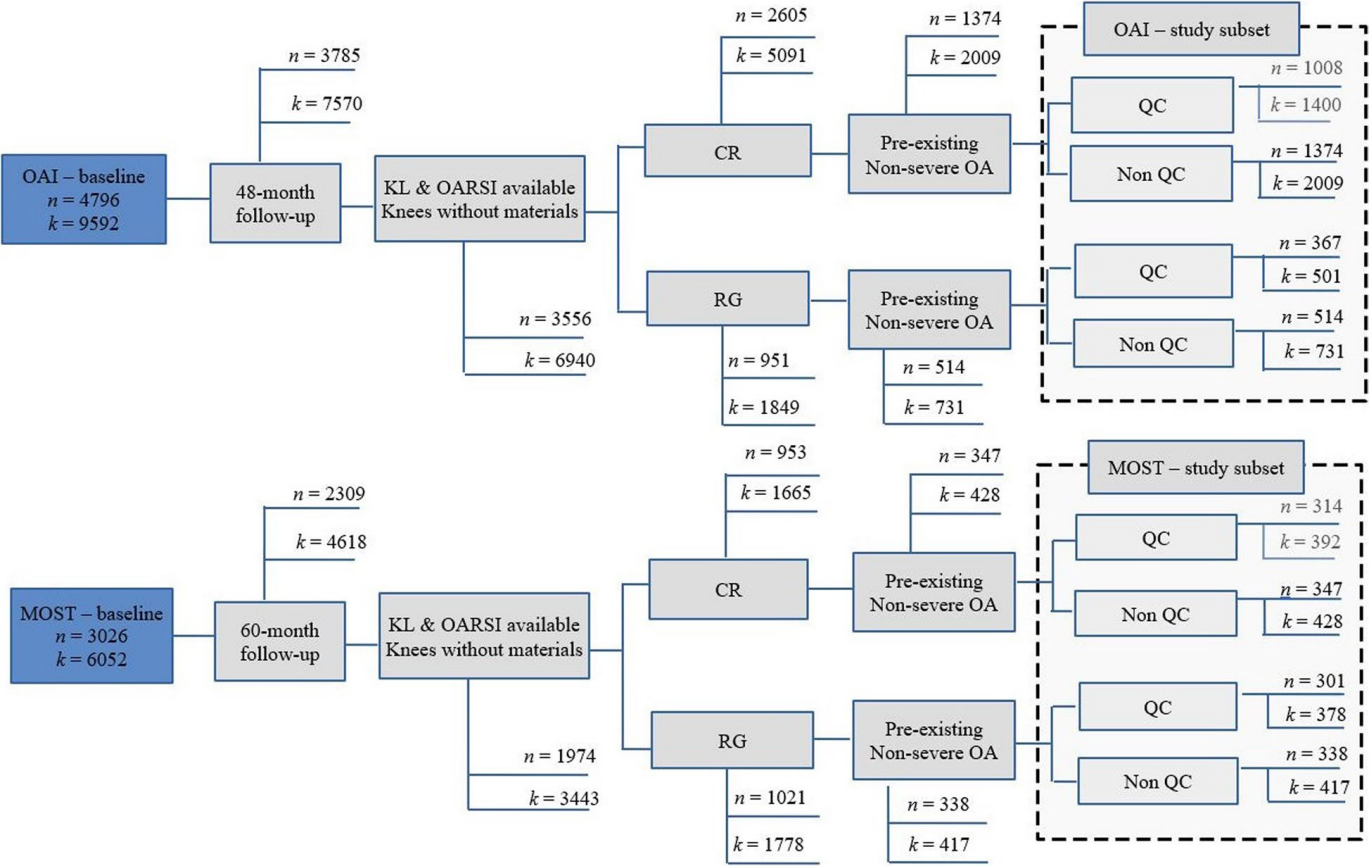
Flowchart illustrating the selection of study subjects from the OAI and MOST datasets (*n* is the number of patients and *k* is the corresponding number of knee radiographs at baseline). From the OAI and MOST initial database, selected sub-datasets were considered, including OA patients with 2 ⩽ KL<4 at baseline, with and without quality control conditions

In order to evaluate the effect of the quality of images on the performance of the predictive models, each of the two datasets (CR and RG) was further divided into two groups according to the quality of the corresponding radiographs. In the first non-quality-controlled (nonQC) group, all radiographs were included except those showing materials (such as metallic materials, prostheses, and screws) in the subchondral zone, whereas in the second quality-controlled (QC) group, exclusion criteria also included radiographs with exposure problems (Fig. 1) in addition to those imposed in the nonQC group. The aim of this exclusion was to avoid the disturbances of these artifacts in the calculation of TBT parameters. This grouping strategy led to four sub-datasets, namely QC-CR, nonQC-CR, QC-RG, and nonQC-RG. As a result of the inclusion/exclusion criteria previously described, 2740 knees (425 cases, OAI) and 845 knees (297 cases, MOST) were judged as eligible for this study. Figure 2 shows the number of subjects and knees for each sub-dataset. The characteristics of selected OA cases and controls are summarized in Tables 1 and 2 for OAI and MOST cohorts, respectively.

**Table 1:** Characteristics of the patients and knees in the OAI subset with different image modalities in the study

	Baseline			48 months		
	Controls	Cases	Total	Controls	Cases	Total
**nonQC-CR**	(*k*=1723)	(*k*=286)	(*k*=2009)	(*k*=1723)	(*k*=286)	(*k*=2009)
Age (years)	62.3 (±9.0)	62.4 (±8.1)	62.3 (±8.8)	66.3 (±9.0)	66.4 (±8.1)	66.3 (±8.1)
BMI (kg/m^2^)	29.4 (±4.6)	30.6 (±4.7)	29.6 (±4.7)	29.3 (±4.8)	31.0 (±5.0)	29.6 (±4.9)
Gender						
F	59%	51%	58%	−	−	−
M	41%	49%	42%	−	−	−
Medial JSN grade						
0	741	41	782	741	0	741
1	617	116	733	617	21	638
2	365	129	494	365	119	484
3	0	0	0	0	146	146
Lateral JSN grade						
0	1421	270	1691	1394	264	1658
1	171	12	183	133	10	143
2	131	4	135	136	11	147
3	0	0	0	60	1	61
**nonQC-RG**	(*k*=592)	(*k*=139)	(*k*=731)	(*k*=592)	(*k*=139)	(*k*=731)
Age (years)	62.9 (±9.1)	62.9 (±8.1)	62.4 (±8.8)	66.3 (±9.0)	66.9 (±8.1)	66.4 (±8.1)
BMI (kg/m^2^)	29.9 ( ± 5.2)	31.0 (±5.1)	30.1 (±5.2)	29.9 (±5.4)	31.4 (±5.6)	30.2 (±5.5)
Gender						
F	67%	63%	36%	−	−	−
M	33%	37%	34%	−	−	−
Medial JSN grade						
0	245	23	268	245	0	245
1	208	44	252	208	9	217
2	139	72	211	139	53	192
3	0	0	0	0	77	77
Lateral JSN grade						
0	498	134	632	489	134	623
1	48	2	50	35	1	36
2	46	3	49	39	3	42
3	0	50	0	29	1	30
**QC-CR**	(*k*=1419)	(*k*=228)	(*k*=1647)	(*k*=1419)	(*k*=228)	(*k*=1647)
Age (years)	62.7 (±9.0)	62.7 (±8.2)	62.7 (±8.9)	66.7 (±9.0)	66.7 (±8.2)	66.7 (±8.2)
BMI (kg/m^2^)	29.3 (±4.6)	30.4 (±4.6)	29.5 (±4.6)	29.3 (±4.8)	30.7 (±4.9)	29.5 (±4.8)
Gender						
F	59%	47%	57%	−	−	−
M	41%	53%	43%	−	−	−
Medial JSN grade						
0	620	31	651	620	0	620
1	496	90	586	496	15	511
2	303	107	410	303	91	394
3	0	0	0	0	122	122
Lateral JSN grade						
0	1164	219	1383	1142	215	1357
1	146	7	153	114	7	121
2	109	2	111	112	6	118
3	0	0	0	51	0	51
**QC-RG**	(*k*=407)	(*k*=94)	(*k*=501)	(*k*=407)	(*k*=94)	(*k*=501)
Age (years)	62.0 (±8.9)	62.4 (±8.6)	62.1 (±8.9)	66.01 (±8.9)	66.4 (±8.6)	66.1 (±8.6)
BMI (kg/m^2^)	29.8 (±5.3)	30.5 (±5.4)	29.9 (±5.3)	29.9 (±5.6)	30.9 (±5.8)	30.1 (±5.6)
Gender						
F	71%	72%	71%	−	−	−
M	29%	28%	29%	−	−	−
Medial JSN grade						
0	184	16	200	184	0	184
1	134	29	163	134	7	141
2	89	49	138	89	34	123
3	0	0	0	0	53	53
Lateral JSN grade						
0	341	89	430	335	89	424
1	35	2	37	23	1	24
2	31	3	34	27	3	30
3	0	0	0	22	1	23

*k* the number of knees

“−” means no changes compared to baseline. Values for age and BMI are represented as mean (±standard deviation)

**Table 2:** Characteristics of the patients and knees in the MOST subset with different image modalities in the study

	Baseline			48 months		
	Controls	Cases	Total	Controls	Cases	Total
**nonQC-CR**	(*k*=306)	(*k*=125)	(*k*=431)	(*k*=306)	(*k*=125)	(*k*=431)
Age (years)	63.2 (±7.7)	62.8 (±7.7)	63.1 (±7.7)	68.2 (±7.7)	67.8 (±7.7)	68.1 (±7.7)
BMI (kg/m^2^)	31.7 (±6.0)	33.3 (±6.7)	32.2 (±6.3)	31.7 (±6.0)	33.8 (±6.6)	32.3 (±6.2)
Gender						
F	67%	62%	65%	−	−	−
M	33%	38%	35%	−	−	−
Medial JSN grade						
0	126	14	140	126	0	126
1	124	45	169	124	11	135
2	56	66	122	56	38	94
3	0	0	0	0	79	76
Lateral JSN grade						
0	216	116	333	202	114	316
1	53	6	59	36	5	41
2	37	3	40	34	3	37
3	0	0	0	34	3	37
**nonQC-RG**	(*k*=244)	(*k*=172)	(*k*=416)	(*k*=244)	(*k*=172)	(*k*=416)
Age (years)	64.4 (±7.6)	63.12 (±8.2)	63.8 (±7.9)	69.4 (±7.6)	68.1 (±8.2)	68.8 (±8.2)
BMI (kg/m^2^)	30.9 (±5.3)	32.2 (±6.7)	31.4 (±5.9)	30.8 (±5.6)	32.6 (±7.3)	31.5 (±6.4)
Gender						
F	68%	55%	63%	−	−	−
M	32%	45%	37%	−	−	−
Medial JSN grade						
0	101	13	114	101	0	101
1	98	80	178	98	10	108
2	45	79	124	45	72	117
3	0	0	0	0	90	90
Lateral JSN grade						
0	208	168	376	187	161	348
1	22	3	25	17	5	22
2	14	1	15	24	6	30
3	0	0	0	16	0	16
**QC-CR**	(*k*=269)	(*k*=107)	(*k*=376)	(*k*=269)	(*k*=107)	(*k*=376)
Age (years)	63.4 (±7.7)	62.9 (±7.7)	63.2 (±7.7)	68.4 (±7.7)	67.9 (±7.7)	68.2 (±7.7)
BMI (kg/m^2^)	31.2 (±5.7)	32.7 (±5.4)	31.6 (±5.6)	31.1 (±5.6)	33.3 (±5.7)	31.7 (±5.7)
Gender						
F	65%	57%	63%	−	−	−
M	35%	43%	37%	−	−	−
Medial JSN grade						
0	116	11	127	116	0	116
1	109	40	149	109	8	117
2	44	56	100	44	34	78
3	0	0	0	0	65	65
Lateral JSN grade						
0	191	100	291	179	100	279
1	49	4	53	32	2	34
2	29	3	32	31	3	34
3	0	0	0	27	2	29
**QC-RG**	(*k*=205)	(*k*=124)	(*k*=329)	(*k*=205)	(*k*=124)	(*k*=329)
Age (years)	64.7 (±7.7)	63.0 (±7.7)	64.0 (±7.7)	69.7 (±7.7)	68.0 (±7.7)	69.0 (±7.7)
BMI (kg/m^2^)	30.9 (+5.4)	32.9 (±7.2)	31.7 (±6.2)	30.9 (±5.8)	33.4 (±7.8)	31.8 (±6.7)
Gender						
F	76%	67%	72%	−	−	−
M	24%	33%	28%	−	−	−
Medial JSN grade						
0	91	11	102	91	0	91
1	78	58	136	78	9	87
2	36	55	91	36	50	86
3	0	0	0	0	65	65
Lateral JSN grade						
0	173	122	295	153	115	268
1	19	1	20	17	5	22
2	13	1	14	20	4	24
3	0	0	0	15	0	15

*k* the number of knees

“−” means no changes compared to baseline. Values for age and BMI are represented as mean (±standard deviation)

### Definition of OA progression

Patients with or without OA progression were selected using the following definitions: OA progressors (cases) included patients with non-severe KOA (KL grade 2 ≤ KL ≤ 3) at baseline and with an increased mJSN grade (ΔmJSN >0) over the predefined control period (48 months and 60 months for OAI and MOST cohorts, respectively. ΔmJSN denotes the difference between OARSI mJSN grades at baseline and check points. OA non-progressors (controls) included patients with non-severe KOA at baseline and a constant mJSN grade (ΔmJSN = 0) over the predefined control period.

### Regions of interest (ROI)

**Fig. 3:**
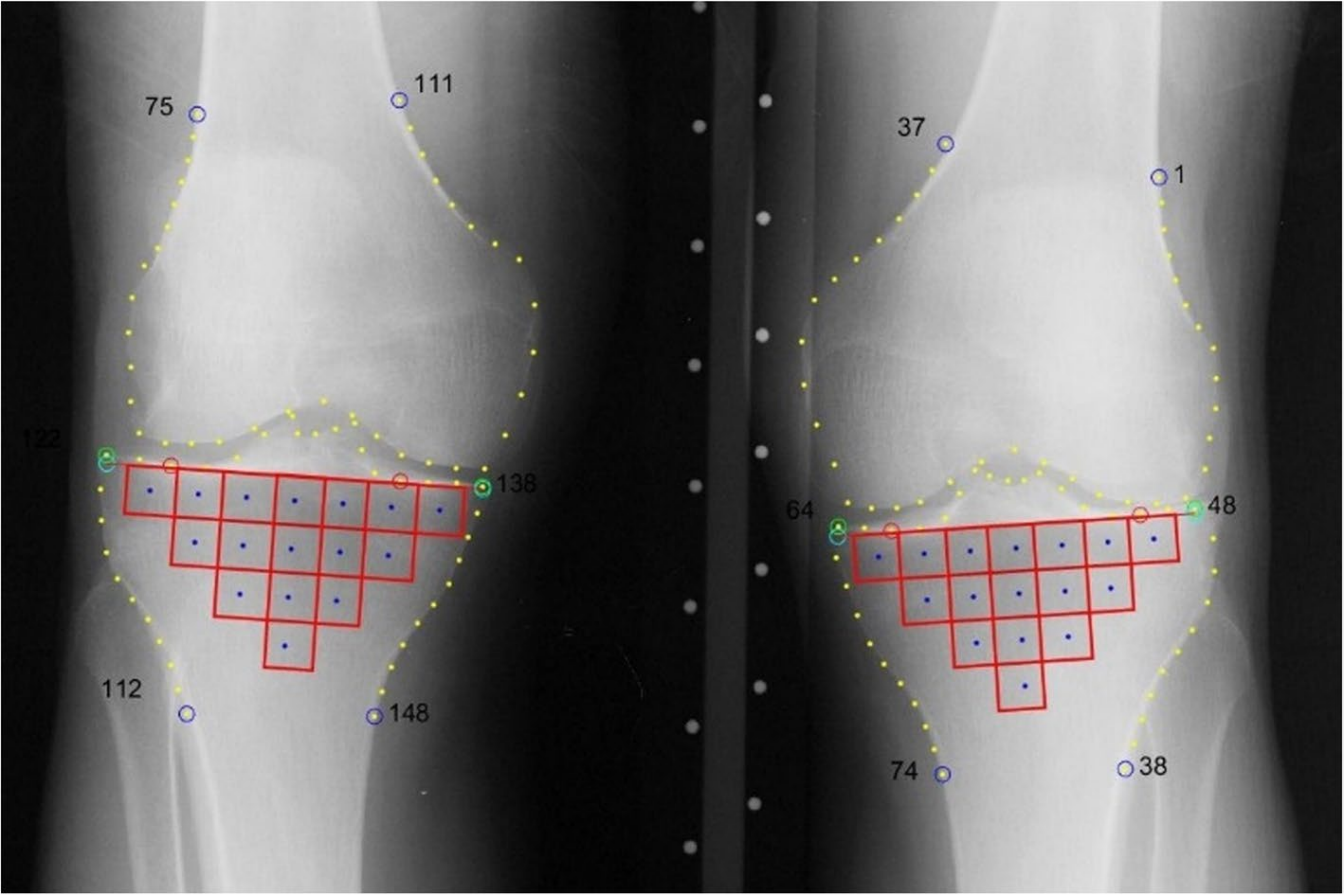
Knee trabecular bone mapping using Bone Finder software for ROI selection. Dots are the anatomical markers automatically defined by Bone Finder. Each patchwork is defined by 16 squared ROIs

A patchwork construction technique using a semi-automatic method to extract the ROIs has been previously described [10]. In the current study, in order to extract the trabecular bone ROIs, a fully automatic approach, thanks to the BoneFinder [19] software, was used to delimit the femoral and tibial bone edges. The patchwork consists of 16 ROIs mapping the whole tibial trabecular area (Fig. 3). Our algorithm firstly uses BoneFinder to identify the rough position of the bone in the image and then outline 148 points of the tibial and femoral contours. For the left knee, points 48 and 64 mark the lateral and medial extremities of the tibia, respectively. For the right knee, the medial extremities of the tibia are identified by the points 122 and 138, respectively. Secondly, the algorithm approximates the tibial subchondral baseline as the line going through these anatomical points. Thirdly, this line is used to determine the orientation and size of the 16-ROI patchwork under the cortical plates. The square ROI dimensions were proportional to the knee width defined as the distance between the outer tibial margins. In our sub-dataset, radiographs presented different pixel spacing ranging from 0.1 to 0.2 mm, and the average ROI side length was 73 ± 18 pixels (10.1 ± 0.9 mm), ranging from 7 to 13 mm.

### Texture analysis

Fractal analysis consists in assigning a fractal dimension (FD) and other fractal characteristics to a dataset [11].

Several methods have been developed to measure the FD of a signal including the well-known technique of fractal signature analysis (FSA) [20], the Whittle estimator (WhE) [7], and the quadratic variation method (VAR) [9, 10]. These three different fractal analysis methods provided consistent results in their capacity to predict OA progression [10]. In the current study, the VAR method, used by Janvier et al. [9], was retained for our experiments.

As reported earlier [9], the cut-off scale was observed around 500 mm on the empirical variograms and two fractal parameters were extracted: μFD and mFD corresponding to the texture complexity computed for the two micro (μ-scale) and the milli (m-scale) scales of observation under 400 mm and above 600 mm, respectively. Four TBT parameters (microscopic scale: horizontal μFD, vertical μFD, and macroscopic scale: horizontal mFD, vertical mFD) were computed in the 16 ROIs, resulting in 64 descriptors.

### KL grading using Siamese neural networks

A Siamese neural network (SNN)-based method proposed by Tiulpin et al. [26] was used to estimate the probability distribution of the KL grades of baseline radiographs included in our study, in the objective to propose a fully automatic KOA progression prediction model. An SNN is a class of neural network architectures that contain two or more subnetworks sharing the same configuration. SNNs are known to be robust to class imbalance, which is usually the case in medical applications [2, 21]. A full description of the used SNN-based method can be found in [25, 26].

### Statistical analysis

Logistic regression was used to predict KOA progression. Several statistical models were developed involving not only clinical covariates and radiological scores but also TBT-based parameters:

- Model_1: cov
- Model_2: cov+TBT
- Model_3: cov+mJSN+lJSN
- Model_4: cov+KL
- Model_5: cov+KLprob
- Model_6: cov+lJSN+mJSN+TBT
- Model_7: cov+KLprob+TBT
- Model_8: cov+KLprob+lJSN+TBT
- Model_9: covPlus+KLprob+lJSN+TBT

where lJSN denotes lateral joint space narrowing, cov denotes the traditional clinical covariates (age, gender, and BMI), and covPlus denotes the cov parameters accompanied with additional clinical data (race, WOMAC pain, and history of injury).

The TBT-CNN model (Model_8) includes baseline TBT, KLprob, lJSN, and cov. The KLprob was computed as the linear combination of the five probabilities of the KL grades predicted by the CNN-based model.

In Model_8, the mJSN was not included, due to the high correlation between the baseline mJSN and KLprob grades.

To avoid overfitting problems, all the models were evaluated using a 10-fold cross-validation repeated 300 times. Each model was evaluated using the AUC of the receiver operating characteristic (ROC) as a global performance criterion. The model classification accuracy (ACC), the probability that a random example is correctly classified, was also computed to investigate the relevance of different models. An ACC is defined as the ratio of the number of correct predictions relative to the total number of predictions.

All statistical analyses were performed using the R Statistical tool (version 3.6.3) including the packages MASS (for stepwise AIC optimization), Caret (for the cross-validation training), and the pROC (for pROC curves and comparisons). Comparisons between the models were based on the ROC curves using the Delong method [6].

In order to reduce the number of parameters before training the prediction models, a backward selection of the TBT parameters (64 variables) was automatically performed using the Akaike Information Criterion (AIC) [1] as an iterative criterion. At each iteration, the AIC removes one parameter and preserves the most efficient parameter(s) to limit overfitting effects.

## Results

### Performance comparison

**Fig. 4:**
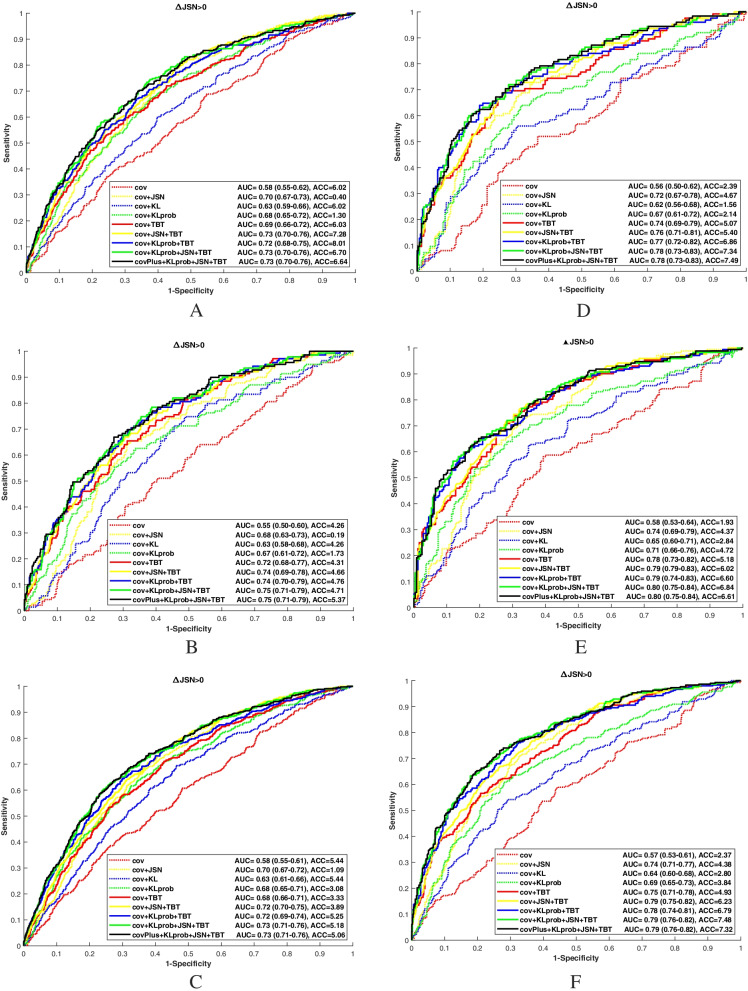
ROC curves obtained for the OA progression prediction. Data from the OAI-nonQC-CR (**A**), RG (**B**), and CR&RG (**C**) sub-cohorts and from the MOST-nonQC-CR (**D**), RG (**E**), and CR&RG (**F**) sub-cohorts. QC and nonQC denote quality control and non-quality control, respectively. CR and RG denote computed radiographs and digitized X-ray films, respectively

The cov and the JSN scores at baseline are presented in Table 1 for the OAI dataset and in Table 2 for the MOST dataset. The ROC curves of the 8 models were calculated using data from nonQC-OAI and MOST sub-datasets (Fig. 4). The models’ AUC values using all considered sub-datasets are summarized in Table 3.

**Table 3:** Summary of AUC values of the 8 models: data from OAI and MOST datasets

	Model_1	Model_2	Model_3	Model_4	Model_5	Model_6	Model_7	Model_8
**OAI-QC**								
CR	0.58	0.69	0.71	0.63	0.69	0.72	0.72	0.75
RG	0.50	0.73	0.68	0.60	0.67	0.67	0.75	0.75
RGCR	0.56	0.69	0.71	0.63	0.69	0.69	0.72	0.74
**OAI-nonQC**								
CR	0.58	0.69	0.70	0.63	0.68	0.72	0.72	0.73
RG	0.55	0.72	0.68	0.63	0.67	0.74	0.74	0.75
RGCR	0.58	0.68	0.70	0.63	0.68	0.72	0.72	0.73
**MOST-QC**								
CR	0.56	0.75	0.74	0.64	0.68	0.78	0.78	0.79
RG	0.58	0.80	0.74	0.65	0.68	0.80	0.80	0.81
RGCR	0.57	0.75	0.75	0.65	0.68	0.78	0.78	0.79
**MOST-nonQC**								
CR	0.56	0.74	0.72	0.62	0.67	0.77	0.77	0.78
RG	0.58	0.77	0.74	0.65	0.71	0.79	0.79	0.80
RGCR	0.57	0.75	0.74	0.64	0.69	0.78	0.78	0.79

In OAI and MOST datasets, Model_1 was not predictive of OA progression (AUC < 0.6). The combination of cov with TBT or KLprob (Model_2 or Model_5 respectively) improved the prediction to a level comparable with that obtained by the combination of cov with JSN (Model_3).

In the MOST dataset, Model_2 was predictive of JSN progression (AUC≥0.74) and significantly better than Model_4 which combines cov and baseline KL (AUC≤0.65); Model_2 outperformed Model_5 (*p* = 0.021); Model_2 significantly improved the prediction compared to Model_3, especially in the RG subset (*p* = 0.017); and Model_3 significantly outperformed Model_5 (*p* < 0.03) in all scenarios regardless of the acquisition modality and image quality.

Model_5 showed a significantly better AUC than Model_4, in all cases (*p* < 0.02 in OAI and *p* < 0.03 in MOST datasets). Model_7 achieved a similar performance with AUCs up to 0.75 (*p* > 0.2) in the OAI dataset and up to 0.80 (*p* > 0.05) in the MOST dataset. The AUCs of Model_7 were significantly better than those of Model_3, especially in the OAI RG subset (*p* < 0.004) and in the MOST dataset (*p* < 0.02). Model_6, which combines cov, JSN, and TBT, previously proposed by Janvier et al. [10], achieved a similar performance.

In all different scenarios, the proposed TBT-CNN model (Model_8) significantly improved the AUC compared to the Model_3 (*p* < 0.003) in the OAI dataset and (*p* < 0.02) in the MOST dataset. Model_8 increased the AUC up to 0.75 in the OAI dataset and 0.81 in the MOST dataset. Model_8 significantly outperformed Model_6 and Model_7 in the OAI CR and CR&RG subsets (*p* < 0.003) and in the MOST CR&RG subsets, regardless of the image quality. The same observation held when considering the MOST-nonQC-RG subset (*p* < 0.05). Furthermore, Model_8 had a good accuracy (ACC > 0.8) in the OAI dataset and (ACC > 0.7) in the MOST dataset. With the additional clinical covariates (race, WOMAC pain, and history of injury) used in Model_9, the results showed no improvement on the prediction performance compared to the proposed model (Model_8) in both OAI and MOST datasets.

### Performance comparison with respect to acquisition modality

In terms of the acquisition modality, no significant differences in AUCs of the 8 models were found with regard to the three different scenarios (CR, RG, and CR&RG) (*p* > 0.1), in both OAI and MOST datasets.

### Performance comparison with respect to image quality

Results showed that the image quality (QC and nonQC) had no statistically significant effect on the performance of the 8 models (*p* > 0.2) in the OAI dataset and (*p* > 0.4) in the MOST dataset. Thus, quality control is not a discriminating determinant of KOA progression prediction.

### The prediction performance of models trained on one dataset and tested in another dataset

Model_8 was tested in two scenarios. In the first scenario, the model was trained on the OAI dataset. The trained model was then used for the prediction of OA progression in the MOST dataset. In the second scenario, the model was trained on the MOST datasets. The trained model was then used for the prediction of OA progression in the OAI dataset.

Results showed the ability of this model trained on one cohort to predict progression in the other cohort with AUC > 0.7 in the CR and CR&RG cases, whatever the quality of the radiographs (Table 4). However, the model trained in the RG subset did not achieve the same performance (AUC < 0.7).

**Table 4:** Results obtained from training on one cohort (OAI/MOST) and testing on another cohort (MOST/OAI)

Modality	Metric	Train. on OAI and validation on MOST		Train. on MOST and validation on OAI	
		NonQC	QC	NonQC	QC
CR	AUC	**0.74 (0.68–0.79)**	**0.73 (0.67–0.79)**	**0.7 (0.67–0.74**)	**0.71 (0.68–0.75)**
	DOR	6.82	5.95	3.42	4.04
	ACC	0.74	0.74	0.77	0.73
RG	AUC	0.64 (0.59–0.70)	0.63 (0.56–0.69)	0.69 (0.64–0.74)	0.59 (0.53–0.65)
	DOR	3.01	4.34	3.36	2.51
	ACC	0.62	0.65	0.74	0.78
CR & RG	AUC	**0.73 (0.70–0.77)**	**0.73 (0.69–0.77)**	**0.71 (0.68–0.74)**	**0.71 (0.68–0.74)**
	DOR	5.82	6.91	4.03	4.04
	ACC	0.7	0.72	0.83	0.82

## Discussion

An important contribution of this study consists in showing that OA prediction models can work with different databases. To the best of our knowledge, the present study is the first to evaluate the capability of combined models, including TBT and CNN-based parameters, to predict KOA progression, in both OAI and MOST datasets. The TBT-CNN model consistently provided the best performance in comparison with the other models [15, 16, 26] not only when training and testing on the same cohort (with AUC up to 0.81) but also when training on one cohort (OAI or MOST) and testing on the other one (MOST or OAI). When testing on another cohort, the TBT-CNN model was always predictive particularly in the CR and CR&RG subsets (AUC ≥ 0.7), which was not the case for the other models.

Our study also included an evaluation of the effect of different acquisition modalities and image qualities on the performance of our combined prediction models.

The TBT-CNN model significantly outperformed the other models, regardless of the quality of the images considered, especially with complete selected OAI and MOST datasets (Fig. 4). The same results were obtained when using the QC- and nonQC-CR sub-datasets of the OAI cohort and the nonQC-RG sub-dataset of the MOST cohort.

The AUC of the TBT-CNN model varied from 0.73 to 0.75 in OAI and from 0.78 to 0.81 in MOST, whereas the AUC of the cov-JSN model achieved a maximum AUC of 0.71 in OAI and 0.75 in MOST (Table 3).

In both cohorts, the results showed that the performance of the TBT-CNN model was invariant with respect to acquisition modality and image quality. Moreover, results showed that the model prediction performance was better when using CNN-based estimations of KL than those measured manually by radiologists in the OAI and MOST datasets. In addition, the performance of the proposed prediction model remained unchanged when adding more clinical data including race, WOMAC pain, and history of injury. Whatever the cohort, the modality of the radiographs, and the quality of the radiographs, the CNN-based estimation of KL grades provided better results than those obtained from a discrete ordinal grading method. An automatic estimation of JSN grades using a CNN-based method [25] might also be of interest to improve the prediction of OA progression.

However, the performance of the prediction model using CNN-based estimations was statistically less significant than when using TBT parameters in the MOST dataset. The performance of the two approaches was similar in the OAI dataset.

Previous studies have demonstrated that the texture analysis of subchondral bone from conventional knee radiographs could be a good indicator of the prediction of knee OA progression [10, 15, 16, 28, 29].

In a recent study by Kraus et al. [15], the use of TBT calculated by the FSA method in combination with other clinical covariates and radiological parameters was investigated to propose a predictive model of OA progression using a large sample of 579 RG&CR radiographs selected from the OAI cohort. They investigated not only the radiographic but also the knee pain progression status over 12 and 24 months. However, the performance of the proposed model was modest (AUC = 0.633 − 0.649).

Involving a much larger dataset of 1124 CR radiographs, Janvier et al. [10] proposed a prediction model that included JSN grades in addition to TBT and cov parameters. In their study, the TBT analysis covered the medial and lateral subchondral bone. This model showed the ability to predict OA progression over 48 months, providing an AUC score of 0.77 using the WhE estimator for the TBT parameters.

### Strengths and limitations

Due to a lack of information in the MOST cohort regarding the JSW, our study took into consideration only the discrete ordinal JSN grades. It would be interesting to consider the use of the continuous JSW values or joint space area (JSA), for which an additional step is required to calculate these values from the selected radiographs.

In the current study, age, sex, and BMI were chosen as clinical predictors. Other predictors of KOA progression such as self-reported previous knee injury and knee pain may also be included in future studies. However, the main focus of this study was to show the ability of image-processing-based models to predict KOA progression, rather than investigating other clinical covariates for KOA progression prediction.

It should be noticed that the duration of the two tested cohorts is not the same (48 months for OAI and 60 months for MOST). Unfortunately, the OAI cohort did not include imaging data at 60-month follow-up, and the MOST cohort did not include imaging data at 48 months. Consequently, the use of time-to-event data analyses was not relevant since the occurrence of KOA progression is more or less a continuous phenomenon. It has been shown, however, that our proposed models provide a good performance in the prediction of KOA progression when trained on one cohort and tested on the other.

The present study has several important strengths. It involves the use of two large datasets. In addition, the proposed model takes advantage of an extensive set of TBT parameters [9, 10] and CNN-based KL grades for the prediction of OA progression. We also evaluated the effect of different image quality and modality scenarios on the performance of the prediction of OA progression. A major contribution of our study is the evaluation using a model trained on one cohort and validated on the other. In this case, the progression prediction models were not only trained on the OAI dataset and tested on the MOST dataset, as proposed by Tiulpin et al. [24], but also trained on the MOST dataset and tested on the OAI dataset, which has never been explored to date. Furthermore, the combination of TBT and CNN-based estimation of KL grades significantly improves the prediction of OA progression. This combination provides mutual information between the evolution of shape surrounding the knee joint space [24–26] and texture variations in the proximal tibial subchondral bone.

## Conclusions

In conclusion, our study has demonstrated the feasibility of using the TBT-CNN model to predict mJSN progression in both OAI and MOST cohorts. This model exhibited a good diagnostic performance regardless of both the acquisition modality and the image quality when the model was trained and tested on the same cohort. Moreover, when trained on one cohort, the TBT-CNN model was able to predict mJSN progression on another cohort in the CR and CR&RG subsets, irrespective of the image quality.

However, further experiments are needed to develop more comprehensive risk assessment models for KOA progression prediction. In particular, other TBT methods such as the Variance Orientation Transform (VOT) [30], FSA, and WhE methods, as well as the automatic calculation of certain radiographic parameters such as JSN, JSW, or JSA scores, could be investigated.

## Data Availability

All data generated or analyzed during this study are included in this published article.

## Abbreviations

ACC: Classification accuracy
AIC: Akaike Information Criterion
AUC: Area under the receiver operating characteristic curve
BMI: Body mass index
CNNs: Convolutional neural networks
cov: Covariates
CR: Computed radiographs
FD: Fractal dimension
FS: Fractal signature
FSA: Fractal signature analysis
JSN: Joint space narrowing
JSW: Joint space width
KJR: Knee joint replacement
KL: Kellgren and Lawrence
KLprob: Estimated Kellgren and Lawrence
KOA: Knee osteoarthritis
lJSN: Lateral joint space narrowing
mJSN: Medial joint space narrowing
MOST: Multicenter Osteoarthritis Study
nonQC: Non-quality-controlled
OA: Osteoarthritis
OARSI: Osteoarthritis Research Society International
RG: Digitized X-ray films
ROC: Receiver operating characteristic
QC: Quality-controlled
ROI: Region of interest
SNN: Siamese neural network
TBT: Bone texture analysis
Var: Quadratic variations
VOT: Variance orientation transform
Whe: Whittle
ΔmJSN: Difference between OARSI mJSN grades at baseline and check points
*μ*FD: FD in microscopic scale
*m*FD: FD in macroscopic scale
